# Clinical therapeutic effects of trastuzumab in HER2-positive breast cancer patients: A protocol for systematic review and meta-analysis

**DOI:** 10.1097/MD.0000000000025685

**Published:** 2021-04-30

**Authors:** Chuanming Tong, Chuan Wang, Kun Yang

**Affiliations:** Department of General Surgery, People's Hospital of Dongxihu District, Wuhan, Hubei, P.R. China.

**Keywords:** breast cancer, efficacy, HER2, safety, trastuzumab

## Abstract

**Background:**

Despite the developments in diagnosis and treatment of HER2-positive metastatic breast cancer, there is a high likelihood in the development of resistance to trastuzumab. In general, HER2-positive patients with deteriorated health face negative clinical outcomes. The present study is conducted to systematically explore the medicinal properties of trastuzumab in HER2-positive breast cancer patients.

**Methods:**

Randomized controlled trials investigating the clinical properties of including trastuzumab to treat HER2-positive breast cancer cases will be sourced by exploring these online-based databases: MEDLINE, BIOSIS, China National Knowledge Infrastructure (CNKI), Cochrane Library, EMBASE, Central Register of Controlled Trials, and WanFang. Two independent authors will screen the literature, gather data, and assess the quality of selected studies. The significance of the relationship between the medical properties of trastuzumab when incorporated to treat HER2-positive breast cancer cases will be evaluated according to the relative risk, mean differences or standardized mean differences, and 95% confidence interval.

**Results:**

The outcomes from this review shall be issued in a journal that will be reviewed by peers.

**Conclusion:**

The conclusions presented in this review will serve as a reference for clinical practitioners and scholars to determine whether trastuzumab is an effective and safety intervention for treating HER2-positive breast cancer patients.

**Ethics and dissemination:**

Since this study is a systematic review of published studies, an ethical approval is not needed.

**Systematic review registration number:**

March 31, 2021.osf.io/wvqkf (https://osf.io/wvqkf/).

## 1 Introduction

According to the 2020 GLOBOCAN, breast cancer is a highly prevalent form of cancer in females with an estimated 2.3 million fresh cases annually.^[1]^ It causes the death an average of 680,000 deaths each year.^[1]^ Nearly 15% to 25% of breast cancer cases are characterized by over-expression of the HER2 protein or amplification of the *HER2* gene, which aggravates the condition, which historically results in negative clinical outcomes.^[2–4]^ Amplification is the underlying process in the over-expression of HER2, and several studies have illustrated a relationship between over-expression of HER2 and a deteriorated overall survival rate and a lesser time to relapse.^[3,5,6]^ Over the last two decades, HER2-directed therapy has improved the survival of metastatic breast cancer patients significantly.^[3]^ Admittedly, treating HER2-positive breast cancer has improved drastically through novel therapeutic approaches. Yet, the median overall survival and progression-free survival for patients with prostate carcinoma prognosis remain poor.^[7]^

Trastuzumab is a humanized monoclonal antibody that resists the extracellular domain of HER2. It became the foremost HER2-targeted therapy accepted for treating HER2-positive breast cancer.^[8,9]^ The combined use of HER2-targeted therapy with chemotherapy, particularly with taxanes, is well established and improves the progression-free survival and overall survival of patients.^[10,11]^ Considering the widespread prevalence of HER2-positive breast cancer, urgent action is needed to devise therapeutic methods. Currently, trastuzumab is officially permitted to be used for HER2-positive breast cancer patients. Therefore, it is crucial to comprehensively understand the efficiency and safety of trastuzumab among this patient population. Therefore, we will plan to conduct this study to assess the medical properties of trastuzumab (including safety and efficacy) when used to care for HER2-positive breast cancer patients.

## 2 Materials and methods

The present study is registered on the Open Science Framework (OSF, https://osf.io). The registration DOI number of this study is 10.17605/OSF.IO/WVQKF. Moreover, this review protocol has been reported according to the PRISMA (Preferred Reporting Items for Systematic Review and Meta-Analyses) guidelines.^[12]^

### 2.1 Eligibility criteria

#### 2.1.1 Types of studies

Each study involved in this review will be randomized, double-blind, parallel-group studies that evaluate the medicinal properties of incorporating trastuzumab in the therapy for HER2-positive breast cancer. All other studies are excluded, which involves observational study, case studies, and non-randomized control studies.

#### 2.1.2 Types of participants

The participants were females with confirmed diagnosis of HER2-positive breast cancer, participant of all ages are included, menopausal condition or hormone receptor condition, or evaluable disease as per the criteria of World Health Organization.

#### 2.1.3 Types of interventions and comparisons

The experimental group received only trastuzumab, or combined with other forms of therapy, such as chemotherapy, hormonal therapy, and targeted agents. The comparative group was administered an identical regimen utilised in the intervention group deprived of trastuzumab. There will not be any limitations on the duration of trials.

#### 2.1.4 Types of outcome measures

##### 2.1.4.1 Primary outcomes

Overall survival on intention-to-treat examination and progression-free survival.

##### 2.1.4.2 Secondary outcomes

1. Complete response rate;
2. Additional toxicities (defined and graded as per the World Health Organization toxicity criteria);
3. Relapse in central nervous system;
4. Therapy-related fatalities;
5. Life standard;
6. Adverse events.

### 2.2 Search methods

#### 2.2.1 Electronic searches

The online-based databases including MEDLINE, BIOSIS, Cochrane Library Central Register of Controlled Trials, EMBASE, China National Knowledge Infrastructure (CNKI), and WanFang are databases that will be explored for randomized controlled trials that evaluate the efficacy and safety of using trastuzumab as a medication strategy for HER2-positive breast cancer. Each database mentioned above will be searched from their starting date to March 2021. There are no limitations on language and publication time.

#### 2.2.2 Searching other resources

Besides the main online databases, this study will search Google scholar, ClinicalTrials.gov (www.ClinicalTrials.gov), and bibliography of each primary study and review article to obtain other related studies.

#### 2.2.3 Search strategy

The search strategy outlined is used: (“breast carcinoma” OR “breast cancer” OR “breast tumour” OR “breast tumor” OR “breast neoplasm”) AND trastuzumab∗ AND (“randomized controlled trial” OR “randomized clinical trial” OR “RCT” OR “randomized”).

### 2.3 Data collection and analysis

#### 2.3.1 Selection of studies

**Figure 1:**
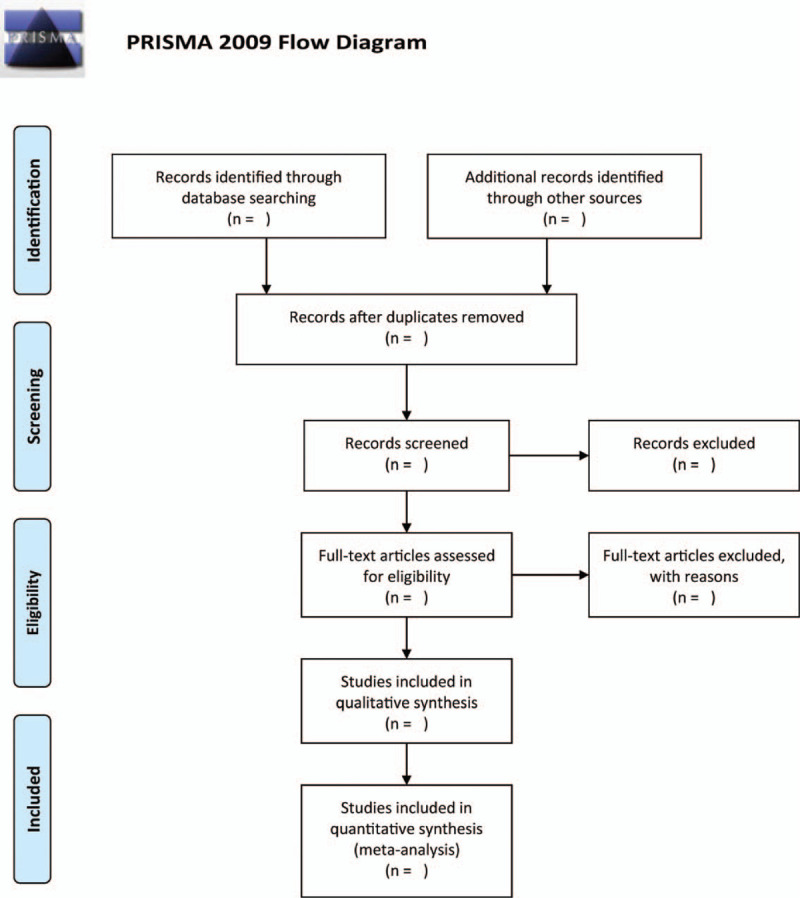
The research flowchart.

In order to select studies, two authors will conduct autonomous selection of literature as per the eligibility criteria that was designed earlier. If a difference in opinion occurs, it shall be determined via conversation, or through consultation with another researcher. The titles and abstracts of the studies shall be scrutinized to remove copies and other unrelated studies. The selection process is illustrated in a PRISMA flow chart (Fig. 1).

#### 2.3.2 Data extraction

For each study selected for inclusion, a minimum of two independent researchers will extract the required information. Afterwards, the data is imported into Microsoft Excel. The included information will comprise the name of author, published year, diagnosis criteria, criteria for suitability, treatment specifics and controller interventions, intervention period, lung functionality, and outcome markers. All disagreements are fixed through discussion, or by consulting a third independent author.

#### 2.3.3 Assessment of study quality

Two independent researchers will assess the quality of the studies involved as per the standards drawn in the Cochrane Collaboration's tool.^[13]^ All differences shall be fixed via conversation or by consulting another autonomous author. This study will evaluate the bias risk in each included study under these aspects: bias in selection, bias in detecting, bias of reporting, bias existing in performance, bias of attrition, and other possible origins of bias. We grade all the origins of bias individually under three levels: “High risk,” “Low risk,” or “Unclear risk.”

#### 2.3.4 Measures of treatment effect

In the present study, the analysis of dichotomous variables is done using the relative risk and for continuous variables the analysis is done using the mean difference or standardized mean differences with its 95% confidence intervals. All statistical analysis is performed with RevMan 5.3 software (Cochrane, London, UK).

#### 2.3.5 Assessment of heterogeneity

The statistical heterogeneity amongst the involved papers shall be determined with the aid of the *I*^2^ test and a general Chi-squared statistic^[14]^. In the case, value of *P* is >.1 and *I*^2^ is <50%, the result corresponds to minor heterogeneity, the fixed-effects model is employed to pool statistics^[15]^; meanwhile, if value of *P* is <.1 and *I*^2^ is >50% indicates substantial heterogeneity, in which case the random-effects model will be employed to pool the statistics.^[16]^

#### 2.3.6 Assessment of reporting biases

This study will use an Egger's test and funnel plots to evaluate any probable publication bias when there are more than 10 studies included.^[17,18]^

#### 2.3.7 Sensitivity analysis

Studies with high bias risk and unclear data will be removed to perform a sensitivity analysis to examine the stability and robustness of the findings in this review.

## 3 Ethics and dissemination

The present systematic review will not require an ethical approval.

## 4 Discussion

Admittedly, previous studies have reported of the potential clinical therapeutic effects of trastuzumab when incorporated in the care of HER2-positivie breast cancer. Having said so, the results are rather controversial. Besides, no previous similar study has systematically evaluated the efficacy and level of safeness of trastuzumab when used as a therapeutic approach for HER2-positive breast cancer patients. Therefore, this systematic review will conduct an analysis to evaluate the efficiency and safeness of trastuzumab when used for providing care for HER2-positive breast cancer. The outcomes of our review are a reference for clinical practitioners and scholars to determine whether trastuzumab is an effective and safe clinical decision to enhance the therapeutic strategies to help HER2-positive breast cancer patients.

## Author contributions

**Conceptualization:** Chuanming Tong, Chuan Wang.

**Data curation:** Chuanming Tong.

**Formal analysis:** Chuan Wang.

**Funding acquisition:** Chuanming Tong, Kun Yang.

**Methodology:** Chuanming Tong.

**Project administration:** Chuanming Tong.

**Resources:** Chuanming Tong, Kun Yang.

**Software:** Chuan Wang.

**Supervision:** Kun Yang.

**Validation:** Chuan Wang.

**Visualization:** Chuan Wang.

**Writing – original draft:** Chuanming Tong, Chuan Wang.

**Writing – review & editing:** Chuanming Tong.
